# Clinical, therapeutic and prognostic characteristics of de novo metastatic breast cancer in Cameroon

**DOI:** 10.3332/ecancer.2026.2092

**Published:** 2026-03-12

**Authors:** Berthe Sabine Esson Mapoko, Etienne Atenguena, Abdel Nasser Nsangou Moun, Esther Dina Bell, Lionel Tabola, Dominique Anaba, Anne Sango, Rachel Tayou

**Affiliations:** 1 Faculty of Medicine and Biomedical Sciences, University of Yaoundé I, Yaoundé 337, Cameroon; 2 Faculty of Medicine and Pharmaceutical Sciences, University of Douala, Douala 2701, Cameroon; 3 Bafoussam Regional Hospital, Bafoussam 995, Cameroon; 4 Faculty of Medicine, University of Buea, Buea 63, Cameroon; 5 Faculty of Medicine, University of Dschang, Dschang 96, Cameroon

**Keywords:** breast cancer, de novo metastasis, overall survival, prognostic factors, Cameroon, Sub-Saharan Africa, resource-limited setting

## Abstract

**Introduction:**

The dilemma of the incurability of metastatic breast cancer has driven therapeutic advances aimed at prolonging survival. However, access to these innovative treatments remains a significant challenge in low-income countries. Consequently, a diagnosis of de novo metastatic breast cancer (dnMBC) may be perceived as a diagnosis of imminent death. We aimed to analyse the clinical, therapeutic and prognostic characteristics of dnMBC in a Cameroonian context.

**Methodology:**

We conducted a cross-sectional, descriptive study with retrospective data collection from 116 patients with dnMBC followed in two cancer reference hospitals in Yaoundé, Cameroon, between 2020 and 2022. Data were analysed using SPSS version 25 and Excel 2019.

**Results:**

Of 1,006 confirmed breast cancer cases, 116 were dnMBC (prevalence: 11.53%). The mean age was 47.4 ± 12.1 years, and males accounted for 1.72% of the sample. Pathologically, the predominant subtype was invasive ductal carcinoma (92.2%; *n* = 83), with hormone-sensitive hormone receptor+/HER2 human epidermal growth factor receptor 2 - tumours being the most frequent among those with available immunohistochemistry (IHC) (58%; n = 18). The disease was often polymetastatic (69.8%), with the most common sites being the lungs (72.4%) and liver (40.5%). Treatments were mainly systemic (53.2%), sometimes combined with surgery (43%). The median overall survival was estimated at 24 months (95% CI = 14.21–33.78).

**Conclusion:**

Survival outcomes for dnMBC remain poor, limited by the lack of full access to optimal care, including systematic IHC testing. A more effective multidisciplinary approach to the disease and the factors affecting survival is needed for optimal utilisation of available therapeutic regimens.

## Background

Breast cancer is the most common cancer worldwide and the leading cause of cancer-related death among women. According to the World Health Organisation, there were approximately 2,261,419 new cases of breast cancer and more than 684,996 deaths among women globally in 2020 [1]. In Cameroon, the same report estimated 4,170 new breast cancer cases and 2,108 deaths in the same year, representing a high case fatality ratio of approximately 50.55% [1]. This elevated mortality is largely attributable to the presence of metastatic disease, as Stage IV breast cancer is consistently associated with poor survival [2].

A diagnosis of de novo metastatic breast cancer (dnMBC) refers to the discovery of synchronous metastatic sites at the time of initial staging [3]. It is estimated that approximately 3%–6% of newly diagnosed breast cancer patients in developed countries have distant metastatic disease at diagnosis, while this incidence is estimated to be between 10% and 30% in developing countries [4]. In Cameroon, this incidence was estimated at 12.21% in 2013 [5]. Globally, dnMBC represents 20%–50% of all metastatic breast cancer cases and about 1%–7% of all metastatic breast cancer cases in patients younger than 40 years [6, 7].

The prognosis for patients with metastatic breast cancer remains difficult to predict and depends on various factors, some related to the patient and others to the disease itself [8]. Despite improved treatment options, metastatic breast cancer remains incurable [9–11], though its prognosis has improved. The median survival for metastatic breast cancer has increased from 18–24 to 30–36 months over the last decade, corresponding to a 5% gain in 5-year overall survival [8]. For de novo metastatic cancers, this median survival increased from 20 to 26 months between 1988 and 2011 to 38 months in 2017 [3, 12], with the 5-year overall survival estimated at 32.6% in 2017 [3]. This has led to the disease being considered a chronic condition [13]. These therapeutic innovations are attributed to progress in molecular characterisation, hormonal therapy, targeted treatments and innovative surgical techniques, such as metastasectomy [14].

However, these advances are generally not accessible in low-income countries due to their high cost [15]. In Cameroon, the minimum wage was estimated at 41,875 XAF per month in April 2023 [16], compared to 261,000 XAF in European countries [17]. The absence of universal health coverage for cancer patients means that care is paid for out-of-pocket in 70.4% of cases [18]. In this context, the discovery of dnMBC could be tantamount to a diagnosis of imminent death due to the difficult access to innovative therapies. However, given the lack of specific data, the reality may be different. We, therefore, aimed to analyse the clinical, therapeutic and survival characteristics of dnMBC patients in a Cameroonian context.

## Materials and methods

We conducted a descriptive and analytical cross-sectional study with retrospective data collection. The study was carried out in the Medical Oncology Departments of the two cancer reference hospitals of Yaoundé in Cameroon, the Yaoundé General Hospital and Yaoundé Central Hospital. The study period spanned 3 years, from 1 January 2020 to 31 December 2022.

Inclusion criteria were defined as any patient file with a histologically or cytologically confirmed diagnosis of breast cancer during the study period, with metastatic lesions discovered via an appropriate workup at the time of initial diagnosis (Stage IV de novo).

Sampling was non-probabilistic, based on the consecutive and exhaustive recruitment of patients meeting the inclusion criteria. The minimum sample size of 165 patients was calculated using the Schwartz formula: *N* = [E2.p.q] / *I*^2^.

We identified breast cancer patients from outpatient registries and then consulted their files stored in the hospital archives. After reviewing the files, we selected those that met our inclusion criteria. Data were recorded on a standardised questionnaire (data collection sheets). We collected data on breast cancer risk factors (age at diagnosis, gender, number of pregnancies, hormonal contraception, family history of breast cancer, alcohol and tobacco consumption), cancer characteristics (histological type, histoprognostic grade, immunohistochemistry (IHC)), number and location of metastases, treatment and survival.

The standard workup used to stage the disease and detect metastases typically included computed tomography (CT) scans or chest X-ray, abdominal/pelvic ultrasound, for patients with cost limitations, following national guidelines. Bone scintigraphy was not available. Brain magnetic resonance imaging were reserved for specific situations due to financial limitations.

We collected data on whether surgery was performed. For dnMBC patients, surgical intervention was categorised based on the intent documented in the medical record, which was predominantly palliative (e.g., local tumour control for bleeding, ulceration or toilet) rather than curative resection of the primary tumour.

Data were entered and analysed using SPSS version 25 and Excel 2019. Central tendency parameters (mean, mode, median) and dispersion (standard deviation) were used to describe continuous variables. Categorical variables were described using percentages. The comparison of qualitative variables was done using the chi-squared test. The Kaplan-Meier method was used to estimate average survival times, and the survival curves were compared using the Log-rank test. Prognostic factors were determined using a Cox model. A *p*-value of <0.05 was considered statistically significant.

Ethical clearance was obtained (N° 0202/UY1/FMSB/VDRC/DAASR/CSD of the 05/13/2023). The data collected were anonymous, and the confidentiality of all information was ensured. Patients were involved in the design and dissemination plans of our research.

## Results

We identified and reviewed 1,288 patient files where breast cancer was suspected at either the Yaoundé General Hospital or the Yaoundé Central Hospital between January 2020 and December 2022. Of these, 1,006 confirmed breast cancer cases were included, and only 116 were dnMBC, representing an overall prevalence of 11.53%.

### Patient characteristics

The age group between 45 and 65 years was the most represented (47.4%; *n* = 55), with a mean age of 47.4 ± 12.1 years. Of the 116 dnMBC patients, 02 were male. Nulliparous patients accounted for 11.2% of the cohort. Twenty-one patients had a family history of cancer, with 38.1% of these being first-degree relatives (Table 1).

### Cancer characteristics and metastasis sites

The diagnosis was cytological in 26 (22.4%) patients and histological in the other 90. When histological examination was performed, the most frequent histological type was invasive ductal carcinoma (92.2%). More than half of the patients were classified as SBR II (57.8%).

IHC was performed in only 33 patients (28.4%). The ‘Luminal A/B’ group, with a positive hormone receptor (HR) status and negative human epidermal growth factor receptor 2 (HER2) status, was the most frequent (58%; *n* = 18) among the 31 conclusive results (Table 2).

The disease was often polymetastatic (69.8% had two or more sites), with the most frequent sites of secondary localisation being the lungs (72.4%) and liver (40.5%) (Table 2).

### Treatment patterns

Of the 116 dnMBC cases, only 79 received specific treatment. The majority of treated patients (53.2%) received systemic treatment alone, and in 43% of cases, it was a combination of surgery and systemic treatment (Table 3). As this was dnMBC, the surgical intervention was predominantly for palliative intent (local tumour control) and not for curative resection.

A total of 77 patients received systemic treatment, always including chemotherapy as a first-line therapy. The anthracycline-based protocol was the most frequently prescribed. 44.1% of patients received 2 treatment lines, and aromatase inhibitors dominated hormone therapy prescriptions (53.8%; *n* = 07). Only two patients received targeted therapy (Trastuzumab). More than half of the patients in our study (67.6%) were adherent to the prescribed systemic treatments.

### Overall survival

**Figure 1.**
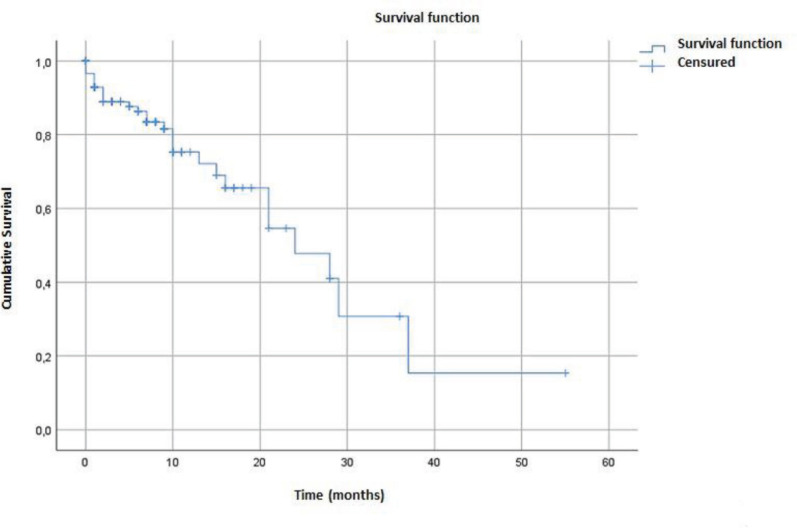
Overall survival of dnMBC patients.

**Figure 2.**
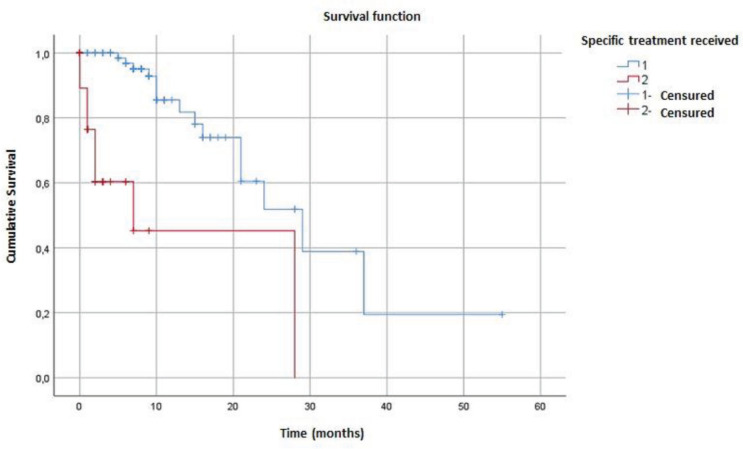
Comparison of overall survival curves of dnMBC patients who received specific treatment.

The median overall survival in our cohort was estimated at 24 months (95% CI = 14.21–33.78) (Figure 1). Survival was better in patients who received specific treatment (29 months) compared to those who did not (7 months) (*p* < 0.001) (Figure 2).

## Discussion

The prevalence of dnMBC in our series was 11.53%. Other studies conducted in our context found a prevalence of metastatic breast cancer at diagnosis of 12.21% in 2015 and 25% in 2018 [5]. These prevalences, while different, corroborate the estimate made by Daily *et al* [4], who found that the incidence of dnMBC in developing countries was between 10% and 30%.

The mean age of patients with de novo metastatic cancer in our series was 47.4 ± 12.1 years, with the age group between 45 and 65 years being the most represented. These results are comparable to those from Madagascar [19], Nigeria [20] and Mali [21], which found similar mean ages, reinforcing the observation that breast cancer affects a younger population in the African context.

The most represented age groups in their series were between 40 and 60 years [19, 20, 21]. In Cameroon, Mapoko *et al* [22] described similar results: an age group of 45–55 years being most represented, with a mean age of 52.27 ± 12.2 years. In general, the threat that breast cancer represents to young women has been consistently reported among Africans, including African Americans [23, 24]. It is, therefore, not surprising that a younger age predominates in our dnMBC population.

### Cancer characteristics

When histological examination was performed, invasive ductal carcinoma was the most represented histological type (92.2%; *n* = 83), and the tumour was very often poorly differentiated (SBR ≥ II in 79.8%). This prevalence is consistent with the findings of many other studies [15, 19, 20, 21, 22].

In our study population, only 33 patients (28.4%) had IHC performed, a critical limitation in a context where treatment often hinges on HR/HER2 status. Among the patients tested, the ‘Luminal A/B’ group was the most frequent (58%; *n* = 18). This result aligns with that presented by Zhao *et al* [25] in China. This low rate is similar to that observed in a Nigerian study [20], highlighting the persistent challenge in resource-limited settings. The unavailability of systematic IHC testing likely leads to a suboptimal use of hormonal therapy (like Tamoxifen or Aromatase Inhibitors) as a first-line treatment for Luminal-positive patients who cannot afford the test, ultimately impacting survival.

### Metastasis sites and treatment

We observed a polymetastatic rate of 69.8%. The most frequent secondary localisation sites were in the lungs (72.4\%) and liver (40.5%). The fact that bone metastases were the fourth most frequent site, differing from some international literature [20, 26–28], may be related to the limitation of the radiological workup used in our centers (lack of systematic, high-resolution CT/positron emission tomography scans or even widespread bone scintigraphy for all patients), which may lead to the under-detection of asymptomatic bone lesions.

Only 79 patients out of the 116 included in our cohort received specific treatment. The 43% rate of patients receiving a combination of surgery and systemic treatment must be interpreted within the context of dnMBC, where surgery is typically performed for palliative intent (local complication management) rather than a curative approach to the primary tumour. This is consistent with guidelines for managing advanced disease. The reliance on anthracycline-based chemotherapy and the low usage of targeted therapy (only two patients received Trastuzumab) and hormone therapy reflect the financial barriers to accessing innovative and targeted treatments [15, 29].

### Prognosis and limitations

The median overall survival in our population was estimated at 24 months (95% CI = 14.21–33.78). This aligns with historical data for dnMBC (20–26 months between 1988 and 2011 [12]), but is significantly lower than the survival achieved in high-income countries (38 months in 2017 [3]). This marked difference underscores that despite local efforts, patient care remains limited by a lack of adequate financial, material, infrastructural and human resources, as demonstrated by the high out-of-pocket costs for essential medicines [29].

### Study limitations

During our work, we faced certain limitations. First, this was a retrospective study, limiting data completeness due to incomplete or lost files, which could have affected the determination of survival. Second, the low rate of IHC testing (28.4%) is a major limitation, restricting the accuracy of our biological profile and prognostic factor analysis. Third, the potential for under-detection of asymptomatic bone metastases due to the limitations of our standard radiological workup (non-systematic use of advanced imaging) might have artificially lowered the rank of bone as a metastatic site. Finally, this study was conducted in two centers considered to be centers of excellence for cancer care, and therefore, may not reflect the reality throughout the entire country.

## Conclusion

The prevalence of dnMBC was 11.53%. The average age of the patients was 47.4 ± 12.1 years. Pathologically, it was mainly invasive ductal carcinoma (92.2%) and hormone-sensitive HR+/HER2- cancers (58%). Metastases developed primarily in the lungs (72.4%) and liver (40.5%). The median overall survival was estimated at 24 months (95% CI = 14.21–33.78). This survival was better in patients who received specific treatment (29 versus 07 months; *p* < 0.001). Improving access to fundamental diagnostic tools like systematic IHC is critical to optimise first-line treatment and improve survival outcomes in this context.

## Conflicts of interest

The authors declare no conflicts of interest.

## Funding

We received no funding for this study.

## Author contributions

Study conception and design: BSEM, EA and ANNM. Data collection: ANNM. Data analysis and interpretation: ANNM, BSEM and EA. Manuscript writing: ANNM, EA and BSEM. Manuscript review: BSEM, ANNM, EA, EDB, LT, AS, RT and PN. All authors have approved the final version of the manuscript. Study guarantor: BSEM.

## Figures and Tables

**Table 1.** Patient characteristics.

Items	*N* = 116	Percentage (%)
**Age (years)**		
≤45	53	45.7
46–**65**	55	47.4
>65	8	6.9
**Risk factors**		
Sex		
Female	114	98.28
Male	2	1.72
**Nulliparous**		
Yes	13	11.2
No	103	88.8
**Hormonal contraception**		
Yes	6	5.2
No	110	94.8
**Family history of breast cancer**		
Yes	21	18.1
No	95	81.9
**Alcohol consumption**		
Yes	29	25
No	87	75
**Tobacco consumption**		
Yes	11	9.5
No	105	90.5

**Table 2.** Cancer characteristics.

Items	*N*	Percentage (%)
**Histological type (*n* = 90)**		
Invasive ductal carcinoma	83	92.2
Invasive lobular carcinoma	2	2.2
Other	5	5.6
**Histoprognostic grade (*n* = 90)**		
Grade I	18	20
Grade II	52	57.8
Grade III	20	22.2
**IHC (*n* = 33)**		
Luminal non-HER2+	18	54.4
Triple negative	8	24.2
HER2+ HR+	3	9
HER2+ HR-	2	6.2
Inconclusive	2	6.2
**Number of metastasis sites**		
1	35	30.2
2–3	68	58.6
4–5	12	10.3
>5	1	0.9
**Metastasis sites**		
Lung	84	72.4
Liver	47	40.5
Pleura	46	39.7
Bone	35	30.2
Lymph node	31	26.7
Brain	3	2.6
Other	5	4.3

**Table 3.** Therapeutic modalities received.

	Count	Percentage (%)
**Specific treatment received (*n* = 116)**		
Yes	79	68.1
No	37	31.9
**Therapeutic protocols (*n* = 79)**		
Systemic treatment only	42	53.2
Chemotherapy only	33	41.8
Chemotherapy + Hormone therapy	8	10.1
Chemotherapy + Targeted therapy	1	1.3
Surgery + systemic treatment	34	43
Surgery only	2	2.5
Systemic treatment + Radiotherapy	1	1.3
